# Impact of NKT Cells and LFA-1 on Liver Regeneration under Subseptic Conditions

**DOI:** 10.1371/journal.pone.0168001

**Published:** 2016-12-15

**Authors:** Ann-Kathrin Jörger, Lei Liu, Karin Fehlner, Tanja Weisser, Zhangjun Cheng, Miao Lu, Bastian Höchst, Andreas Bolzer, Baocai Wang, Daniel Hartmann, Volker Assfalg, Yoshiaki Sunami, Anna Melissa Schlitter, Helmut Friess, Norbert Hüser, Melanie Laschinger

**Affiliations:** 1 Department of Surgery, Klinikum rechts der Isar, Technische Universität München, Munich, Germany; 2 Institute of Molecular Immunology, Klinikum rechts der Isar, Technische Universität München, Munich, Germany; 3 Carl Zeiss Microscopy, ZEISS group, Munich, Germany; 4 Institute of Pathology, Technische Universität München, Munich, Germany; University of Leeds, Faculty of Medicine and Health, UNITED KINGDOM

## Abstract

**Background:**

Activation of the immune system in terms of subseptic conditions during liver regeneration is of paramount clinical importance. However, little is known about molecular mechanisms and their mediators that control hepatocyte proliferation. We sought to determine the functional role of immune cells, especially NKT cells, in response to partial hepatectomy (PH), and to uncover the impact of the integrin lymphocyte function-associated antigen-1 (LFA-1) on liver regeneration in a subseptic setting.

**Methods:**

Wild-type (WT) and LFA-1^-/-^ mice underwent a 2/3 PH and low-dose lipopolysaccharid (LPS) application. Hepatocyte proliferation, immune cell infiltration, and cytokine profile in the liver parenchyma were determined.

**Results:**

Low-dose LPS application after PH results in a significant delay of liver regeneration between 48h and 72h, which is associated with a reduced number of CD3^+^ cells within the regenerating liver. In absence of LFA-1, an impaired regenerative capacity was observed under low-dose LPS application. Analysis of different leukocyte subpopulations showed less CD3^+^NK1.1^+^ NKT cells in the liver parenchyma of LFA-1^-/-^ mice after PH and LPS application compared to WT controls, while CD3^-^NK1.1^+^ NK cells markedly increased. Concordantly with this observation, lower levels of NKT cell related cytokines IL-12 and IL-23 were expressed in the regenerating liver of LFA-1^-/-^ mice, while the expression of NK cell-associated CCL5 and IL-10 was increased compared to WT mice.

**Conclusion:**

A subseptic situation negatively alters hepatocyte proliferation. Within this scenario, we suggest an important impact of NKT cells and postulate a critical function for LFA-1 during processes of liver regeneration.

## Introduction

The liver is known to play an important regulatory role within the immune defense [1]. Vice versa, the immune system is highly involved in liver regeneration processes as proven by changes in hepatic cytokine and chemokine profiles after partial hepatectomy (PH) [2]. However, the role of NK and NKT cells within regenerative processes remains controversial. On the one hand, NK cells are known to inhibit liver regeneration by the secretion of IFNᵞ, while NK cell depletion causes an increased intrahepatic DNA synthesis and elevated expression of various cyclins after PH [3]. On the other hand, hepatocyte proliferation after PH in the absence of B, T, and NK cells (Rag2^-/-^ᵞc^-/-^ mice) was found to be reduced compared to the situation in which only B and T cells were genetically depleted (Rag1^-/-^) [4]. Analysis with regard to NKT cells showed that their intrahepatic number markedly increased during liver regeneration after PH and that these cells were able to mediate cytotoxicity against regenerating hepatocytes [5]. Activated NKT cells were reported as enhancer of liver damage after PH via TNFα and IFNᵞ [6]. In contrast, activation of NKT cells by α-galactosylceramide after PH correlate with increased hepatocytes proliferation [7].

In the clinical setting, highly efficient liver regeneration is observed after surgery, e.g., upon living liver donation. After PH of the right hepatic lobe, the remaining liver tissue of the donor doubles within 7 days and regains its original weight after 60 days [8]. Moreover, the liver’s unique regenerative capability is of major clinical relevance in patients with primary liver cancer or metastasis that are intended to be resected. The liver is confronted continuously with antigens from the portal vein blood and is able to eliminate these under physiologic conditions. After PH, this ability is affected, and the residual liver tissue is more vulnerable to infections translating into subseptic conditions and finally to sepsis-related liver failure [9]. For instance, in oncologic surgery, simultaneous resection of a colonic tumor and liver metastases remains controversial in view of the risk of bacterial translocation from the colon and consecutive subsepsis and sepsis-induced hepatic failure. In clinical investigations a significant increased complication rate of 38% compared to patients who received sequential resection for liver metastases (20% complication rate) was identified. However, no significance in patient survival was found [10]. Nevertheless, experimental approaches to clarify the impact of subseptic conditions on liver regeneration after PH are sparse.

In the present study, we put an emphasis on the investigation of the influence of NK and NKT cells on liver regeneration under subseptic conditions in a mouse model by PH and low-dose LPS application. An additional aim of the study encompasses the presumption that the presence of LFA-1 is essential for intact hepatic regeneration under low-dose LPS.

## Materials and Methods

### Animals

C57BL/6N mice, aged 10–12 weeks, were purchased from Charles River. LFA-1^-/-^/C57BL/6N mice were described previously [11]. Animal experiments were institutionally approved by the government of Oberbayern (AZ 55.2.1.54-2532-123-11).

### Partial hepatectomy and LPS application

Approximately 70% of the liver was removed from isoflurane-anaesthetized female mice using a modified protocol from Higgins [12]. Briefly, ligation and resection of the major part of the median and entirely left lateral lobe was performed separately using a surgical microscope (Stemi, DV4, Zeiss Microscopy, Jena, Germany). In sham-operated mice, the liver was palpated with a sterile cotton bud. In case of performing PH in presence of low-dose LPS 50μg LPS (O55:B5, Sigma-Aldrich, Steinheim, Germany) per mouse (20g body weight) was administered intraperitoneally immediately after PH or sham operation. At designated time points, mice were analgized and sacrificed under isoflurane anesthesia through punction of the aorta, the remnant liver was perfused with PBS and the liver/body-weight-ratio was calculated as described before [12]. For immunofluorescence staining and RNA-analysis, tissue was frozen in liquid nitrogen. For immune cell isolation, freshly prepared organs were used.

### Serum chemistry assay

Blood was taken from the aorta and serum alanine aminotransferase (ALT) activities were determined using a cobas c system (ALTPM) by Roche/Hitachi (Risch, Switzerland) at the Institute of Clinical Chemistry, Klinikum rechts der Isar, Technical University of Munich.

### Immunofluorescence

For immunofluorescence staining, 5μm frozen liver tissue sections were fixed with 3% PFA and blocked with species-specific serum (Sigma-Aldrich, Steinheim, Germany). Sections were incubated with specific antibodies for 1h at RT. The following antibodies were used: anti-cCaspase3 (Cell Signaling Technologies, Danvers USA), anti-CD3e (145-2C11, Becton Dickinson (BD) Biosciences, Heidelberg, Germany), anti-CD4 (RM4-5, Biolegend, San Diego, USA), anti-CD8a (53–6.7, Biolegend, San Diego, USA), anti-CD68 (AbD Serotec, Düsseldorf, Germany), anti-Ly6G and Ly6C (RB6-8C5, BD Biosciences, Heidelberg, Germany), and anti-Ki67 (MMI, Leica Biosystems, Wetzlar, Germany). Slides were washed and incubated with fluorochrome conjugated second stage antibody and DAPI (Sigma Aldrich, Steinheim, Germany) for 30min. The following second stage antibodies were used: anti-armenian-hamster-IgG-AlexaFluor^®^488, anti-armenian-hamster-IgG-Cy3, anti-rabbit-IgG-DyLight^®^549, anti-rat-IgG-DyLight^®^549 (all Dianova, Hamburg, Germany), and anti-rat-IgG-Alexa Fluor®488 (Life Technologies, Darmstadt, Germany). Stained sections were mounted with ProLong Gold Antifade Reagent (Invitrogen) and analyzed using an Observer.Z1 attached to an AxioCam MRm camera, an Plan-Apochromat 40x/1,3 NA and 100x/1,4 NA oil objective and the AxioVision Software (all Zeiss Microscopy).

### Reverse transcription PCR

Total RNA was extracted from frozen liver tissue according to the manufacturer's instructions using TRI Reagent^®^ (Sigma-Aldrich, Steinheim, Germany). Reverse transcription was performed using the RevertAid First Strand cDNA Synthesis Kit (Fermentas, St. Leon-Rot, Germany). To detect gene expression levels of CCL5, IL-12, TNFα and IL-10 a hydrolysis-probe-assay for quantitative real-time polymerase chain reaction (qRT-PCR) was used. Gene expression of IL23p19 was detected using SYBR^®^-Green-I (Life Technologies, Darmstadt, Germany), the primers for β-actin (5’aaggccaaccgtgaaaagat3’, 5‘gtggtacgaccagaggcatac3’), CCL5 (5‘tgcagaggactctgagacagc3’, 5‘gagtggtgtccgagccat3’), IL-12 (5'tcagaatcacaaccatcagca3', 5'cgccattatgattcagagact3'), IL-6 (5’gctaccaaactggatataatcagga3’, 5‘ccaggtagctatggtactccagaa3’), IL-10 (5‘cagagccacatgctcctaga3’, 5‘tgtccagctggtcctttgtt3’), IL-23p19 (5’gccttggaagcgggcaag3’, 5‘tggccaaagcctgggctc3’), TNFα (5‘tgcctatgtctcagcctcttc3’, 5‘gaggccatttgggaacttct3’) and a LightCycler^®^ 480 II (Roche, Risch, Switzerland) according to manufacturer’s protocol. For the hydrolysis-probe-assay, reactions were processed for 1 cycle (600s) at 95°C, followed by 45 cycles of 10s at 95°C, of 30s at 60°C and of 1s at 72°C, finishing with 1 cycle of 30s at 60°C. For SYBR^®^-Green-I-assay, reactions were processed for 1 cycle (300s) at 95°C, followed by 45 cycles for 10s at 95°C, for 10s at 55°C and for 10s at 72°C, continued by 1 cycle for 5s at 95°C and 60s at 65°C. Expression levels were normalized to endogenous β-actin mRNA.

### Isolation of liver lymphocytes and flow cytometry

Freshly resected liver organ was cut into pieces, homogenized in RPMI/HEPES, and passed through a 100μm cell strainer (BD, Heidelberg, Germany). To enrich for leukocytes, cells were centrifuged at 52g for 3 min, the supernatant was collected and washed. Lymphocytes were enriched using a sucrose gradient. Briefly, 4ml of cell suspension in 40% Easycoll separating solution (Merck-Milipore, Darmstadt, Germany) was overlaid on 3ml of 70% Easycoll separating solution. After centrifugation at 600g for 25 min, the interphase layer containing the lymphocytes was collected, washed, stained with specific antibodies, and analyzed by flow cytometry using a FACSCalibur™ (BD, Heidelberg, Germany) or a SP6800 Spectral Analyzer (Sony Biotechnology, Weybridge, UK) and the software Flow Jo 8.8.2 (TreeStar Inc., Ashland, USA). The following antibodies were used: anti-CD45.2-FITC (clone: 104), anti-NK1.1-APC (clone: NKR-P1C), anti-CD3e-PE (clone: 145-2C11), anti-Thy1.2-APC (all from BD, Heidelberg, Germany), anti-TCRß (clone: H57-597; Biolegend, Fell, Germany), and anti-CD11a-FITC (clone: I21/7; Life Technologies, Darmstadt, Germany). 7-AAD (7-Aminoactinomycin D) was purchased from Biolegend. Cells were stained with αGalCer-loaded CD1d-dextramer according to the manufacturer’s protocol (Immudex, Copenhagen, Denmark).

### Cell lysis and western blotting

Organs were washed in 5% phosphatase inhibitor/PBS (Active Motif, La Hulpe, Begium) and stored at -80°C. Upon defrosting, SDS sample buffer was added and samples were boiled for 5 minutes at 95°C. Lysates were subjected to SDS-PAGE followed by transfer to nitrocellulose membrane. Incubation with primary and HRP-conjugated secondary antibodies allowed protein-detection via ECL western blotting detection reagents (Pierce via ThermoFischer Scientific, Waltham, USA). The following antibodies were used: anti-pSTAT3(Tyr705), anti-SOCS3 (L210), anti-GAPDH (all from Cell Signaling, Danvers, USA) and goat-anti-rabbit-HRP (Jackson ImmunoResearch via Dianova, Hamburg, Germany).

### Statistical analysis

Data were expressed as means ± standard deviation. Statistical differences were analyzed by two-tailed unpaired Student’s t-test or two-way ANOVA-test. In both tests, a difference was considered significant, when the p-value was <0.05. P-values are indicated as follows: *p<0.05, **p<0.01, ***p<0.001, ns = not significant. If not otherwise stated, statistical differences between two groups were calculated to be not significant.

## Results

### Liver regeneration after PH under low-dose LPS

**Fig 1:**
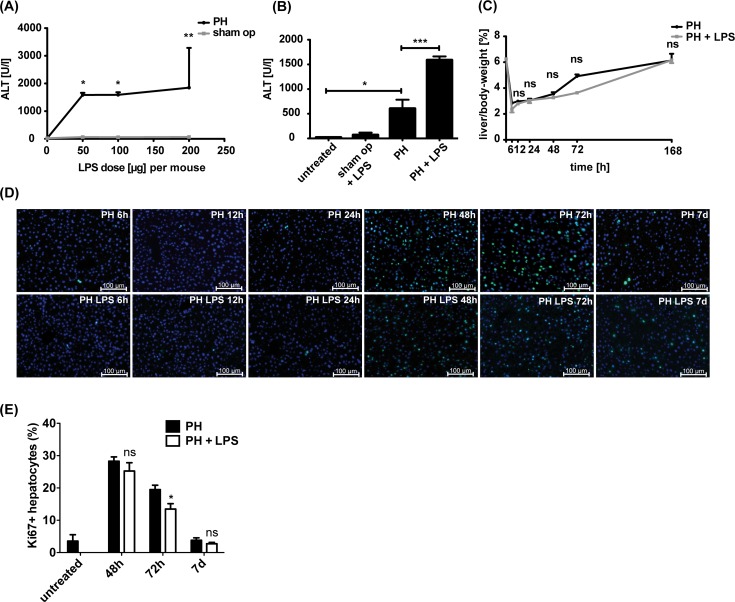
LPS dose-response profile and liver regeneration under low-dose LPS stimulus. WT mice underwent sham-operation or PH followed by intraperitoneal application of different doses of LPS. LPS dosage per 20g body weight is depicted. (A) Quantitative analysis of the liver specific enzyme alanine-aminotransferase (ALT) 24h post surgery (n = 3). (B) ALT serum levels in WT mice 24h after sham-operation or PH in absence or presence of 50μg LPS per 20g body weight are shown (n = 3). (C) Liver/body-weight-ratio in percent of WT mice at different time points upon PH or PH and LPS application. Seven days (168h) post surgery complete liver regeneration was observed. Graph represents mean ± SD from 3 mice for each setting. (D) Hepatocyte proliferation within the liver parenchyma at distinct time points post-PH or PH and LPS. Proliferating hepatocytes were detected by Ki-67 (green) and nuclear DAPI (blue) using immunofluorescence staining of liver cryosections. Liver sections of non-operated mice served as untreated control. Representative images are shown (n = 3 mice). Scale bars indicate 100μm. (E) Percentage of Ki-67 positive hepatocytes in relation to all hepatocytes was quantified. Mean ± SD from at least 5 cryosections for each time point from two mice (n≥2000 hepatocytes) is shown.

The liver not only responds to local LPS during septic shock-induced organ failure but also reacts to broad low-dose LPS exposure of the whole organism [13]. To determine adequate time points and the exact LPS concentration inducing a low-dose liver injury, different concentrations of LPS were injected into wild type (WT) mice after sham-operation or partial hepatectomy (PH), and alanine aminotransferase (ALT) was measured for the amount of liver damage (Fig 1A and 1B). Application of a dosage of 50μg LPS per 20g body weight of the mouse results in an increase in liver transaminases after PH (Fig 1A). Therefore, this concentration was defined as “low-dose” for all further investigations. In contrast, application of different doses of LPS upon sham-operation did not induce a significant amount of ALT (Fig 1A and 1B).

The liver/body-weight-ratio was measured at different time points, and its peak at 72h post-PH indicated that major regenerating processes in the liver occur between 48h and 72h post-PH (Fig 1C). In concordance, we detected the maximum of proliferative Ki67-positive hepatocytes 48h post-PH (Fig 1D and 1E). Compared to PH only, mice receiving PH and low-dose LPS application showed a slightly lower increase in liver/body-weight-ratio (Fig 1C) between 48h and 72h and a significantly reduced number of Ki67-positive hepatocytes at 72h (Fig 1D and 1E). Upon 7 days post-PH as well as PH under subseptic conditions the number of Ki67-positive hepatocytes and the liver/body-weight-ratio were found to be comparable to the preoperative situation indicating complete liver regeneration even in the presence of low-dose LPS. Analyzing liver tissue with regard to apoptosis revealed only few cleaved Caspase3-positive cells at all postoperative time points after PH or PH and LPS (S1 Fig and data not shown). These data suggest, that reduced liver regeneration under subseptic conditions at 48h and 72h post-PH mainly results from impaired hepatocyte proliferation rather than from increased hepatocyte decline. Taken together, our results show that low-dose LPS does not completely hinder liver regeneration upon PH but seems to markedly delay hepatic proliferation 72h post hepatectomy.

### Characterization of innate and adaptive immune cells in liver regeneration under low-dose LPS

**Fig 2:**
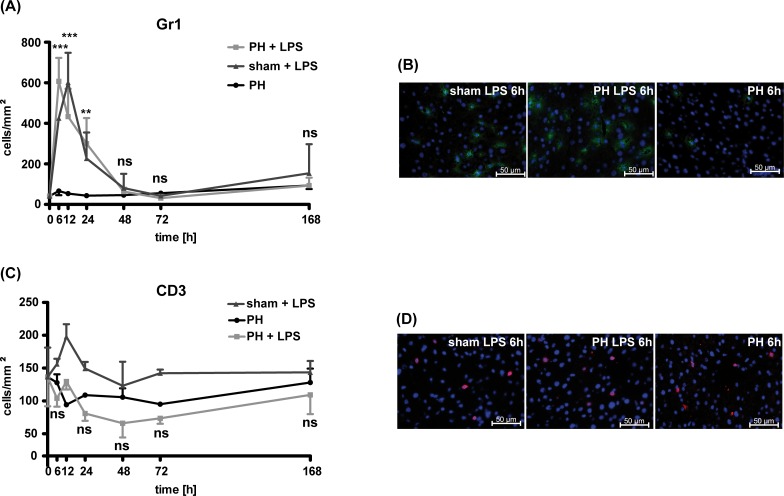
Characterization of immunocompetent cells in the regenerating liver. Number of Gr1^+^ (A) or CD3^+^ (C) immune cells per mm^2^ liver parenchyma were quantified in the regenerating liver at distinct time points after PH, PH plus LPS application or sham-procedure plus LPS application in WT mice. The 0h timepoint represents liver tissue of WT mice (n = 2) without any treatment and before the operation (preoperative situation). Representative images for Gr1^+^ (B, green) or CD3^+^ (D, red) cells within the liver parenchyma 6h after sham procedure plus LPS, PH plus LPS or PH only in WT mice are shown. At least 5 sections per time point and treatment were analyzed. Each value represents the mean ± SD from 3 independent mice per time point. Statistical significances were analyzed by two-way ANOVA-test and refer to the comparison between PH and PH and LPS application. Scale bars indicate 50μm. Gr1^+^ cells in green, CD3^+^ cells in red, nuclei in blue (DAPI).

In order to identify immune cell subsets from the innate and the adaptive immune system that might influence liver tissue regeneration under low-dose LPS, we determined the presence of granulocytes within the liver parenchyma. Under subseptic conditions, a significant number of neutrophil granulocytes could be detected in the liver parenchyma after PH and after sham-operation. However, this recruitment can be ascribed exclusively to the LPS application as it was not detectable after PH only (Fig 2A and 2B).

In contrast to the high numbers of neutrophils being present in liver upon PH plus low-dose LPS at early time points (6-24hrs), we could not detect an increase in T cell numbers when compared to the baseline level preoperatively (Fig 2C and 2D). Following PH under subseptic conditions when compared to PH only, a slight but not significant reduction in the numbers of T cells could be identified. Impaired liver regeneration upon PH in the presence of LPS—in line with reduced numbers of T cells within the liver parenchyma—might point towards a protective role of T lymphocytes during liver regeneration especially under conditions of low-dose LPS.

### LFA-1 influences liver regeneration but not the inflammatory activity in liver parenchyma

**Fig 3:**
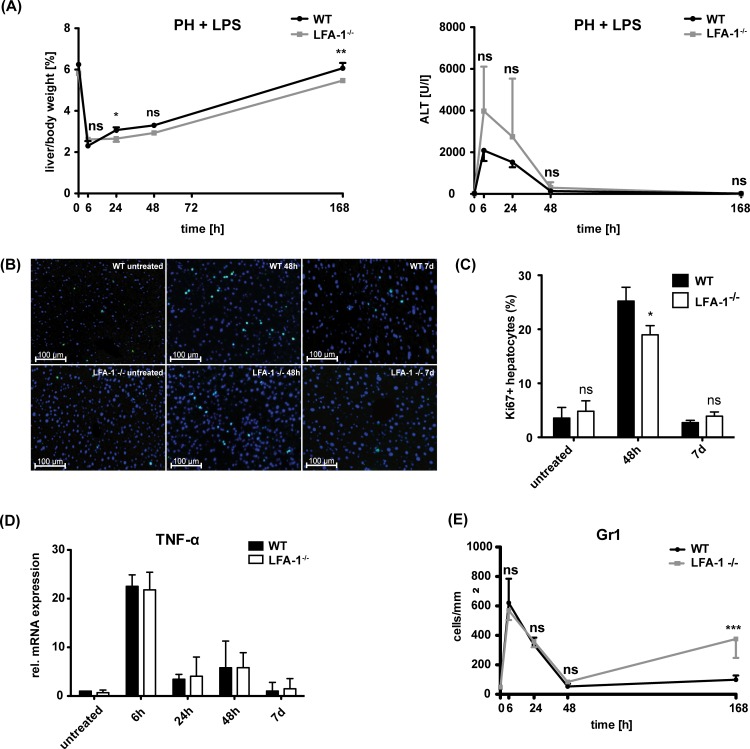
Impact of LFA-1 on liver regeneration. (A) Liver/body-weight-ratio in percent and amount of ALT in the serum of WT or LFA-1^-/-^ mice at different time points upon PH and application of 50μg LPS per 20g body weight. Values represent mean ± SD from 3 mice per time point. Statistical significances were analyzed by two-way ANOVA-test and refer to the comparison between the WT and LFA-1^-/-^ mice. (B) Detection of proliferating Ki67-positive hepatocytes in the liver parenchyma at 48h and 7 days in the liver parenchyma of WT or LFA-1^-/-^ mice. Scale bars indicate 100μm. Ki-67 (green), nuclear DAPI (blue). (C) Mean ± SD of Ki-67 positive hepatocytes in relation to all hepatocytes in percent is shown. At least 5 cryosections for each time point from two mice (n≥2000 hepatocytes) were analyzed. (D) Analysis of TNFα-mRNA upon PH and low-dose LPS in the liver parenchyma of WT and LFA-1^-/-^ mice. Values represent mean ± SD from 3 mice per time point. Statistical differences between WT and LFA-1^-/-^ were tested by two-way ANOVA-test. (E) Quantification of Gr1^+^ neutrophils in the liver parenchyma after PH and LPS application by immunofluorescence staining on liver cryosections. Values represent mean ± SD from at least 5 cryosections for each time point (n = 3 mice). Statistical differences between the WT and LFA-1^-/-^ were tested by two-way ANOVA-test.

In the model of sepsis-mediated liver injury, LFA-1 has been shown to play a major role in the firm adhesion of leukocytes to post-sinusoidal venules within the liver [14]. PH and low-dose LPS application were both applied in LFA-1^-/-^ mice in order to investigate the influence of LFA-1 on liver regeneration under subseptic conditions. During the whole study period, LFA-1-deficient mice presented with a decreased liver/body-weight-ratio in the presence of an inflammatory subseptic stimulus (Fig 3A). The liver/body-weight-ratio of WT mice 7 days after PH was found to be comparable to the preoperative situation, however, in the absence of LFA-1, it did not reach its preoperative level 7 days after PH and LPS application, pointing to an incomplete liver regeneration within this context (Fig 3A). Major proliferation within the liver of WT mice, as detected by Ki67-positive hepatocytes, was found at 48h post-PH and LPS application (Fig 3B and 3C). In contrast, the liver of LFA-1-deficient mice showed significantly less hepatocyte proliferation at 48h (Fig 3B and 3C). In absence of LFA-1, serum ALT levels 24h post-operation under subseptic conditions were higher compared to WT mice, indicating more hepatic damage (Fig 3A). Thus, we investigated whether the reduced regenerative capacity under low-dose LPS application in the absence of LFA-1 might result from impaired immune responses in the liver of these mice. As TNFα is known as a crucial inflammatory mediator, we analyzed TNFα-mRNA in the liver parenchyma of WT and LFA-1-deficient mice receiving PH and low-dose LPS. A remarkable postoperative increase of TNFα-mRNA expression was found at the early time points (6h) and this rapidly declined from 24h post-PH to preoperative levels on day 7 post-PH (Fig 3D). During the whole observation period, no significant differences in hepatic TNFα-mRNA expression was detected in WT compared to LFA-1-deficient mice (Fig 3D). Neutrophils as well as Kupffer cells are known as a major source of TNFα in the liver tissue [15]. In concordance with the comparable expression of TNFα-mRNA, we found similar numbers of Gr1^+^ neutrophils (Fig 3E) and no obvious difference in the amount of CD68^+^ macrophages (data not shown) within the liver parenchyma of WT and LFA-1-deficient mice at early postoperative time points after PH and low-dose LPS. Interestingly, after 7 days a second peak of neutrophil recruitment into the liver parenchyma was detected exclusively in LFA-1-deficient mice.

#### NKT lymphocytes possess a protective role in liver regeneration under low-dose LPS

**Fig 4:**
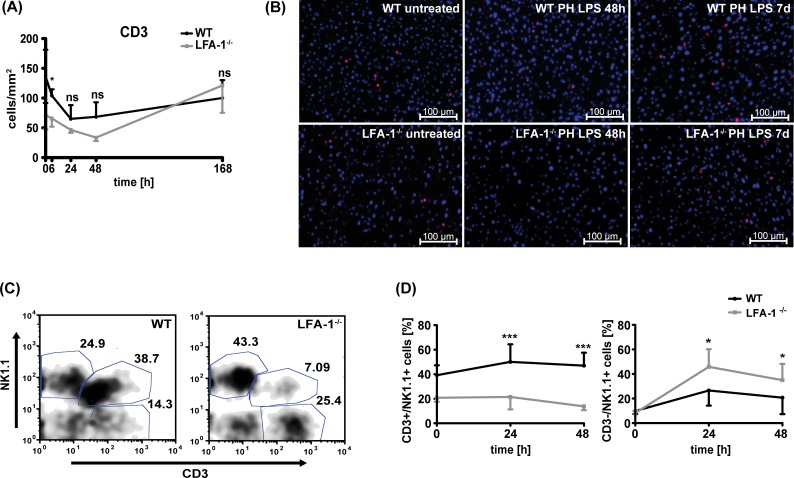
Absence of LFA-1 results in decreased numbers of NKT cells within the regenerating liver. Detection of T cells within the liver parenchyma of WT or LFA-1^-/-^ mice after PH and LPS application. (A) CD3^+^ cells within the liver were quantified on at least 5 sections per group and number of CD3^+^ T cells per mm^2^ tissue was calculated. Data from 3 mice per time point are shown. Statistical differences between WT and LFA-1^-/-^ were tested by two-way ANOVA-test. (B) Representative images 48h and 7d (168h) post-PH and LPS are depicted using immunofluorescence staining of liver cryosections. CD3^+^ T cells appear red and nuclear DAPI blue. Scale bars indicate 100μm. (C, D) Detection of CD3^+^NK1.1^-^ T cells, CD3^-^NK1.1^+^ NK cells or CD3^+^NK1.1^+^ NKT cells in the regenerating liver of WT or LFA-1^-/-^ mice at distinct time points after PH and low-dose LPS using flow cytometry. (C) A representative dot-plot is depicted for gated CD45^+^ leukocytes in a WT or a LFA-1^-/-^ liver 24h post-PH and LPS. (D) Quantitative analysis of CD3^+^NK1.1^+^ NKT cells and CD3^-^NK1.1^+^ NK cells in the liver parenchyma of WT and LFA-1^-/-^ mice. Data show percentage of cells from all leukocytes within the liver parenchyma (mean ± SD) from 4 mice per time point. The significances refer to the difference between WT and the LFA-1^-/-^ mice and were tested by two-way ANOVA.

As our data indicate that loss of LFA-1 does not affect the presence of important elements of the innate immune system in the regenerating liver, we investigated whether recruitment of T cells into the liver parenchyma might be influenced in LFA-1-deficient mice upon PH and low-dose LPS application. In WT controls, the number of T cells in the liver decreased at the early postoperative time points (Fig 4A and 4B). In LFA-1^-/-^ mice, T cell numbers in the hepatic parenchyma were considerably lower in pre- and early postoperative samples up to 48h compared to WT mice (Fig 4A and 4B). This reduced number of T lymphocytes in absence of LFA-1, together with the impaired regenerative capacity, suggests a protective role of T lymphocytes in liver regeneration under subseptic conditions. Circulating T cells were quantified in peripheral blood of untreated mice to exclude the possibility that LFA-1^-/-^ mice have less T cells *per se*. As described before [11], we found even more circulating T cells in LFA-1^-/-^ compared to WT mice (S2 Fig). Interestingly, a marked recruitment of T lymphocytes into the liver parenchyma was detected in LFA-1-deficient mice after 7 days (Fig 4A and 4B). When we analyzed different leukocyte subpopulations isolated from WT livers post-PH and low-dose LPS, we found CD3^+^NK1.1^+^ NKT cells to be recruited to the liver parenchyma 24h and 48h hour post-PH (Fig 4C). In absence of LFA-1, we detected less CD3^+^NK1.1^+^ NKT cells in the liver parenchyma after PH and LPS application (Fig 4C and 4D). At the same time, the percentage of CD3^-^NK1.1^+^ NK cells was markedly increased in LFA-1^-/-^ mice 24h and 48h post-PH under subseptic conditions (Fig 4D). Overall significant less CD45^+^ leukocytes could be detected in the regenerating liver of LFA-1 deficient when compared to WT mice (data not shown). It has been shown that besides NKT cells NK1.1 expression can be detected on activated conventional T cells as well as on non-MHC-restricted type II NKT cells possessing a diverse TCR repertoire. To corroborate the presence of CD1d-restricted type I NKT cells in the regenerating liver, we detected TCRß^+^αGalCer/CD1d-dextramer^+^ liver NKT cells in WT mice and found the number of these cells to be elevated at 48h post PH and LPS application compared to the preoperative situation. In contrast, no substantial increase of TCRß^+^αGalCer/CD1d-dextramer^+^ NKT cells could be detected in the liver of LFA-1^-/-^ mice 48h post PH and LPS (S3A and S3B Fig).

Presence of activated NKT cells has been described to support hepatocyte proliferation and liver regeneration [7]. Thus, the reduced recruitment of NKT cells to the liver parenchyma in the absence of LFA-1 might account for the delayed liver regeneration upon PH and low-dose LPS in LFA-1-deficient mice.

**Fig 5:**
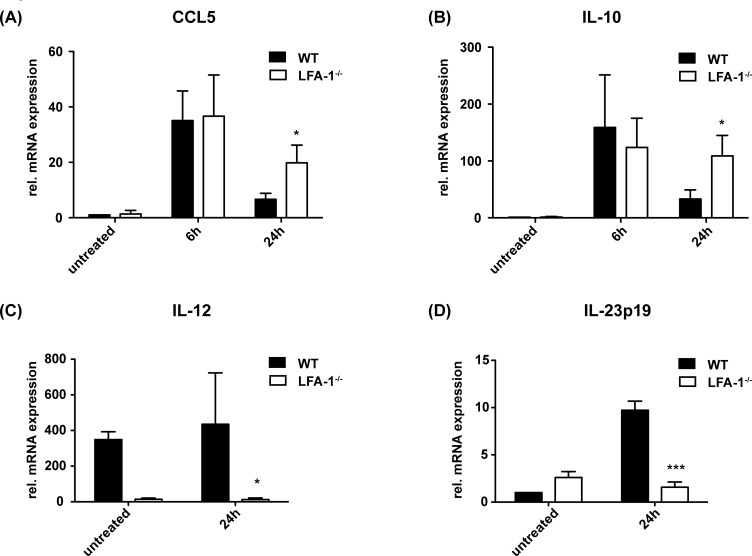
The cytokine milieu in liver parenchyma after PH and low-dose LPS. PH in presence of 50μg LPS per 20g body weight was performed in WT and LFA-1^-/-^ mice and amount of CCL5 (A), IL-10 (B), IL-12 (C) or IL-23p19 (D) cytokine/chemokine-specific mRNA was analyzed by qRT-PCR. The amount of cytokine/chemokine-specific mRNA in the liver tissue of the untreated WT mice was defined as standard value 1. Mean ± SD from 4 mice is depicted. Significances refer to the comparison of the WT and LFA-1^-/-^ mice and were tested by two-way ANOVA.

Chemokines and cytokines have a major impact on specific immune cell recruitment and differentiation, and differentiated effector cells release specific pro- and anti-inflammatory cytokines. Thus, we analyzed the cytokine and chemokine profiles in the liver post-PH and low-dose LPS. CCL5 expression has been demonstrated during liver regeneration [2]. In the hepatic parenchyma of WT as well as LFA-1^-/-^ mice, increased expression of CCL5-specific mRNA could be found in postoperative samples (Fig 5A). At 24h post-PH, CCL5 mRNA significantly increased in LFA-1-deficient compared to the WT mice. As CCL5 is a known NK cell attractor [16], elevated levels of CCL5 in the liver of the LFA-1-deficient mice 24h after PH might account for increased numbers of NK cells. In addition, mRNA of the immunosuppressive cytokine IL10, known to be produced by NK cells during acute and systemic infections [17] and to negatively regulate hepatocyte proliferation [18], was found to be significantly increased in LFA-1^-/-^ compared to WT mice at 24h after PH and LPS application (Fig 5B). Besides its immunosuppressive effect, IL-10 affects the synthesis of IL-12 [19], and IL-12 is well known as a cytokine of indirect NKT cell activation [17]. In the regenerating liver of WT mice, we found that the increased numbers of NKT cells correlate with the considerable amount of IL-12 mRNA (Fig 5C). In contrast, in the absence of LFA-1 NKT cells seem not to be recruited into the liver after PH and low-dose LPS, and consequently, we detected almost no IL-12 mRNA within the liver of these mice (Fig 5C). NKT cells constitutively express the IL-23 receptor [20], and 24h after PH, a noticeable induction of IL-23p19 mRNA was detected in the liver tissue of the WT mice (Fig 5D). Contrariwise but concordant with a reduced number of NKT cells in the liver parenchyma, the expression of IL-23p19 mRNA was significantly lower in the LFA-1^-/-^ mice compared to the WT mice at 24h after PH and LPS application (Fig 5D).

**Fig 6:**
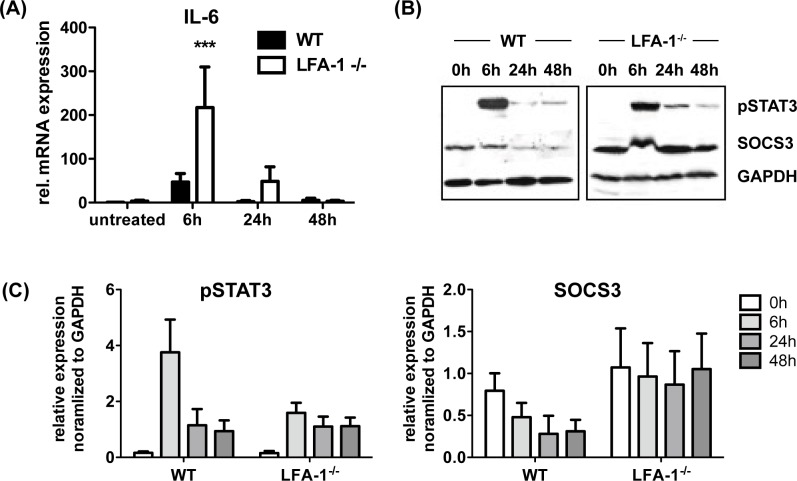
Increased level of IL-6 mRNA in the regenerating liver of LFA-1^-/-^ mice is accompanied by low level of phosphorylated STAT3 (pSTAT3) but increased amount of SOCS3 protein. PH in presence of 50μg LPS per 20g body weight was performed in WT and LFA-1^-/-^ mice and amount of (A) IL-6 specific mRNA within the liver parenchyma was analyzed by qRT-PCR. IL-6 specific mRNA in the liver of untreated WT mice was defined as standard value 1 (mean ± SD from 3 mice). (B) Detection of pSTAT3 and SOCS3 protein in the regenerating liver of WT and LFA-1^-/-^ mice upon depicted timepoint after surgery. GAPDH served as loading control (representative immunoblot). (C) Quantification of results in (B) are depicted as mean ± SD from 3 mice for each timepoint.

As the cytokine IL-6 is well known for its beneficial impact on liver regeneration after partial hepatectomy, we investigated whether impaired liver regeneration in LFA-1^-/-^ mice is accompanied with reduced expression of IL-6 and its downstream signaling target phosphorylated STAT3 (pSTAT3) [21]. Surprisingly, we found 6h after PH and LPS application a significant higher increase in IL-6 specific mRNA in the liver of LFA-1^-/-^ mice when compared to WT mice (Fig 6A). This increase in IL-6 mRNA did not translate into high levels of pSTAT3 protein, but rather seem to correlate inversely with the amount of pSTAT3 protein detected in the regenerating liver of LFA-1^-/-^ mice (Fig 6B and 6C). Recently, suppressor of cytokine signaling 3 (SOCS3) has been demonstrated to negatively regulate STAT3 phosphorylation and hepatocyte proliferation [22]. In concordance with these results, we found 6h post PH and low dose LPS high levels of pSTAT to accompany with reduced expression of SOCS3 protein in the liver of WT mice (Fig 6B and 6C). In contrast, LFA-1^-/-^ mice seem to have more SOCS3 protein prior PH and during liver regeneration, and no major changes in SOCS3 expression could be detected (Fig 6B and 6C).

## Discussion

Bacterial translocation from the colon, e.g. via postoperative intestinal anastomotic leakage, displays a clinically important risk for patients. In cases of simultaneously performed surgical liver resection and colectomy (e.g., for hepatic metastases), the impact of systemically released LPS on liver regeneration is ill defined. Our findings clearly show that LPS delays liver regeneration but does not completely suppress it in a subseptic setting. LPS as a toll-like receptor 4 ligand has been reported to play a central role in the initiation of liver regeneration [23, 24]. Rudich et al. even found reduced hepatocyte proliferation in partial hepatectomized mice after removing LPS from the gut via antibiotic pretreatment [25]. In addition, hepatocyte-specific deletion of NEMO, the regulatory subunit of the NF-κB complex, leads to impaired liver regeneration after partial hepatectomy independent of an inflammation [26]. On the other hand, inhibition of the central inflammatory mediator NF-κB does not result in impaired liver regeneration in mice after partial hepatectomy [26]. A moderate inflammatory stimulus seems to be essential for the initiation of liver regeneration, whereas marked inflammatory reactions have a negative impact [27, 28]. Thus, complete elimination of LPS via antibiotic bowel decontamination [25] prevents the stimulatory effect. Tail vein injection of LPS in rats [29] or intraperitoneally application in mice induces the accumulation of neutrophils in the liver [30]. These cause liver damage by the release of reactive oxygen radicals oxidating stored lipids, or via lysosomal enzymes that destroy hepatocytes by protein degradation. Our data concordantly indicate that neutrophils recruitment to the liver parenchyma cannot be ascribed to the hepatectomy procedure *per se*, but to the presence of LPS. Therefore, we conclude that granulocytes are not directly associated with the regenerative processes in the liver. Moreover, despite neutrophil recruitment to the liver, we did not find a significant elevation of ALT in the blood of sham-operated mice receiving LPS compared to sham operation in absence of LPS. These results emphasize that the presence of neutrophils itself does not necessarily lead to hepatocyte damage.

With regard to CD3^+^ T lymphocytes, we specifically revealed that the amount of T cells present within the regenerating liver does not change significantly after PH only or sham-procedure plus LPS application. In contrast, a marked reduction of T cell numbers in the liver was observed during hepatic regeneration in the subseptic setting. The interaction of the ß2-integrin LFA-1 on the lymphocyte surface and its corresponding receptor on endothelial cells is crucial for leukocyte migration processes [31]. Liver sinusoidal endothelial cells express the LFA-1 ligand ICAM-1 [1]. Its pivotal role in regenerative liver processes was mainly derived from experiments with ICAM-1^-/-^ mice, which revealed a decreased hepatocyte proliferation after PH [15]. In addition, LFA-1 has been demonstrated to control T lymphocyte migration into hepatic tissue [32–34]. Interestingly, a reduced liver damage was found in the absence of LFA-1 after the application of LPS and D-galactosamine in a sepsis model [14, 35]. In contrast, our experiments indicate a beneficial impact of LFA-1 on liver regeneration upon partial hepatectomy under subseptic conditions. Absence of LFA-1 resulted in an increased liver cell damage and impaired regenerative capacity of the liver in LFA-1^-/-^ mice after PH and LPS application. A positive influence of T cells on liver regeneration was postulated by Tumanov et al. [36]. Their data showed a higher mortality of T cell-deficient Rag1^-/-^ mice after PH. T cells might positively modulate liver regeneration by a Lymphotoxin-mediated pathway [36, 37]. While these observations are based on PH only, our data suggest a protective impact of immunocompetent T lymphocytes even in the subseptic setting. Furthermore, reduced T cell numbers in the liver of LFA-1^-/-^ mice seems to be enough to guarantee as much sufficient liver regeneration as needed for survival. Nevertheless, T cell migration is not only restricted to LFA-1/ICAM-1 interaction. In concordance, we found T lymphocytes within the regenerating liver of LFA-1^-/-^ mice. In addition, in presence of inhibitory antibodies directed against LFA-1 and ICAM-1 T cells are still able to attach to sinusoidal endothelial cells in a LFA-1-independent manner [38]. Previous studies and our own data show that although VCAM-1 is not expressed on healthy hepatic vessels, in the regenerating liver inflammatory processes trigger VCAM-1 expression [39, 40].

Our investigations revealed a marked increase in NKT cell counts after PH plus LPS application at the early postoperative time points in the liver parenchyma of WT mice. In contrast, their number was decreased in the absence of LFA-1. The role of NKT cells in liver regeneration is subject of controversial discussion. On the one hand, a cytotoxic effect of NKT cells on regenerating hepatocytes after PH was found [5]. However, other investigations report a positive impact of NKT cells on liver regeneration after stimulation with α-galactosylceramide [7] and their increased release of IL-4 [2], respectively. As we found reduced number of NKT cells to be accompanied by less liver regeneration in LFA-1^-/-^ mice, our data support the hypothesis that LFA-1 positively influences NKT cell recruitment into the regenerating liver and indicate a protective role of NKT cells. This observation includes a contrary reaction of NK cells. NKT cells were found to have an enhanced proliferation in murine hepatic tissue after NK cell depletion [41]. A mechanism is being postulated in which NK and NKT cells compete for the proliferation promoting cytokine IL-15, and consequently, the expansion of one population suppresses the proliferation of the other [41]. This model could explain the antagonistic reaction of NK and NKT cell numbers in the regenerating liver of LFA-1^-/-^ mice during liver regeneration, which we observed. It was previously reported that NK cells especially make use of VCAM-1/VLA-4 and not LFA-1/ICAM-1 to migrate into hepatic tissue in an inflammatory setting [42]. This supports our findings that NKT cells but not NK cells are affected by loss of LFA-1 in the regenerating liver under subseptic conditions.

NK cells, representing almost 30% of all intrahepatic lymphocytes [43], are known to respond to CCL5 [44]. In a model of Concanavalin A-induced acute hepatitis, the treatment of CCR5^-/-^ mice with a monoclonal anti-CCL5 antibody led to markedly reduced hepatocyte damage [45] and a significant reduction in NK cells in the liver parenchyma [16]. Therefore, increased expression of CCL5 in the absence of LFA-1 in our model might account for increased NK cell recruitment into the regenerating liver. Additionally, we found enhanced expression of IL-10 in LFA-1^-/-^ mice after PH and LPS application. Different cellular sources of IL-10 in the context of inflammatory processes are known [46–48], and besides Kupffer cells NK cells were characterized as a strong producer of IL-10 in non-lymphoid tissue during acute systemic infections [17]. Moreover, a crucial role in the LPS-mediated systemic shock is attributed to NK cells [35]. IL-10 is a potent immunosuppressive cytokine acting as a repressor by negatively regulating liver regeneration via a limiting inflammatory response and subsequently tempering hepatic STAT3 activation [18]. The IL-12 production is consecutively limited [17]. IL-12 is well known as a cytokine of indirect NKT cell activation [17]. NKT cells constitutively express the IL-23 receptor [20]. Our results showed a reduced mRNA expression of IL-12 and IL-23 in the absence of LFA-1 that was in line with a reduced number of NKT cells in the liver parenchyma during hepatic regeneration under subseptic conditions. The impaired regenerative capacity of LFA-1^-/-^ mice suggests that NKT cells might positively influence liver regeneration after PH under subseptic conditions.

In addition, we were able to show that an increase in IL-6 levels goes in line with low pSTAT3 and high SOCS3 levels in LFA-1^-/-^ mice 6 hours after PH and LPS application. This finding is supported by work of Riehle et al. showing that SOCS3 negatively regulates STAT3 phosphorylation and hepatocyte proliferation [22]. Although presence of IL-6 has been clearly demonstrated to support liver regeneration, recent data suggests that IL-6-dependent signaling might also negatively regulate hepatocyte proliferation, and these data highlight a potential role of SOCS3 for liver regeneration [20, 49]. Whether NK cells are involved in this scenario is not yet fully understood. Depletion of NK and NKT cells not only impairs hepatocyte proliferation, but also results in a decreased amount of pSTAT3 and surprisingly SOCS3 [50]. In contrary, data from Riehle et al. as well as data from this study show that low pSTAT3 is associated with increased SOCS3 expression, indicating that SOCS3 might be an important negative regulator during liver regeneration [22]. This finding is strengthened by the fact that SOCS3-deficient hepatocytes have also been shown to display an increased activation of STAT3 and ERK1/2 in response to EGF, which itself points to an increased responsiveness to proliferative effects of growth factors, such as EGF [22].

In the present manuscript, we were able to show that a subseptic setting negatively influences liver regeneration. Within this context, an essential role of LFA-1 and a beneficial impact of NKT cells have been identified. These findings might potentially play an important role in clinical decision-making and the development of novel therapy strategies. Firstly, due to a substantial delay of liver regeneration in a sub-septic, LPS-mediated setting, simultaneous operations on the liver and colon cannot be recommended. Secondly, stimulating NKT cells might be a potential therapeutic approach in order to improve liver regeneration after partial hepatectomy, e.g. for oncological reasons or for living liver transplantation.

## Supporting Information

S1 Fig Identification of only few cleaved Caspase 3 (cCaspase3) apoptotic cells in the liver parenchyma post-PH and LPS application. Using immunofluorescence on sections of the liver parenchyma post-PH and LPS, only rare events of cCaspase3-positive apoptotic cells (red) could be detected. Exemplary image depicting a liver section of WT mice at 24h after PH and LPS application suggests that cCaspase3-positive cells (arrow) are not hepatocytes exhibiting characteristic nuclear (blue) shape and size (arrowhead). The scale bar indicates 50μm.
(TIF)

S2 Fig T cell numbers in the blood of the WT and LFA-1^-/-^ mice. Quantification of Thy1.2^+^ T cells within the blood of untreated WT or LFA-1^-/-^ mice using flow cytometry. A representative assay is shown (n = 3).
(TIF)

S3 Fig Reduced numbers of NKT cells within the regenerating liver in absence of LFA-1. (A) Detection of TCRß^+^αGalCer/CD1d-dextramer^+^ cells within the liver of WT or LFA-1^-/-^ mice before (ex vivo) or 48h post PH and LPS using flow cytometry. One representative assay is shown; 20.000 events per dot plot are shown. (B) Quantification of TCRß^+^αGalCer/CD1d-dextramer^+^ NKT cell staining depicted in (A); 0h: n = 4, 24h: n = 3, 48h: n = 3.
(TIF)
