# Supplementary figures and images for: Impact of NKT Cells and LFA-1 on Liver Regeneration under Subseptic Conditions
Source: PLoS One. 2016 Dec 15;11(12):e0168001. doi: 10.1371/journal.pone.0168001 (PMC5158001; doi:10.1371/journal.pone.0168001)

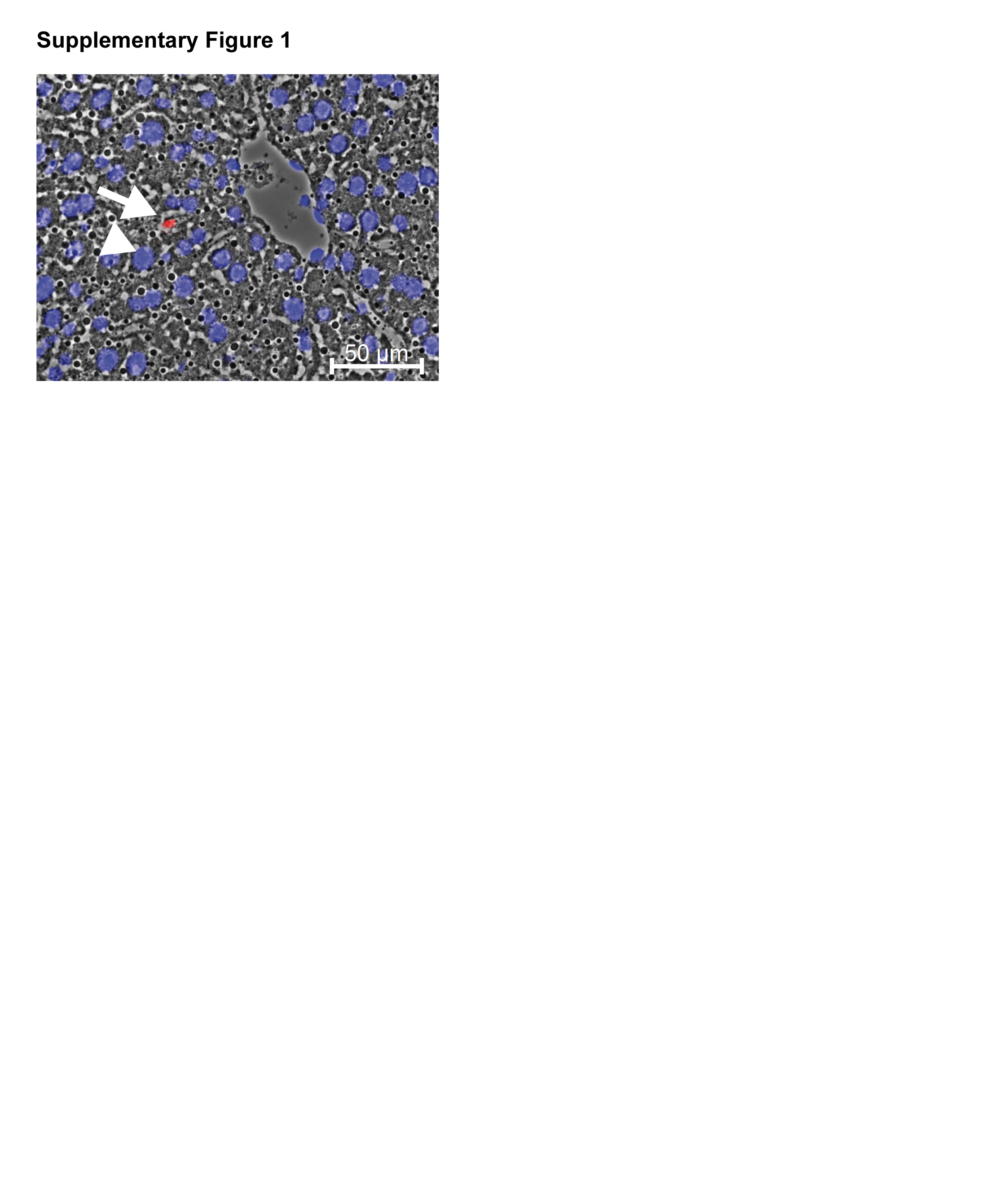

Supplement: S1 Fig — Using immunofluorescence on sections of the liver parenchyma post-PH and LPS, only rare events of cCaspase3-positive apoptotic cells (red) could be detected. Exemplary image depicting a liver section of WT mice at 24h after PH and LPS application suggests that cCaspase3-positive cells (arrow) are not hepatocytes exhibiting characteristic nuclear (blue) shape and size (arrowhead). The scale bar indicates 50μm. (TIF) [file pone.0168001.s001.tif]

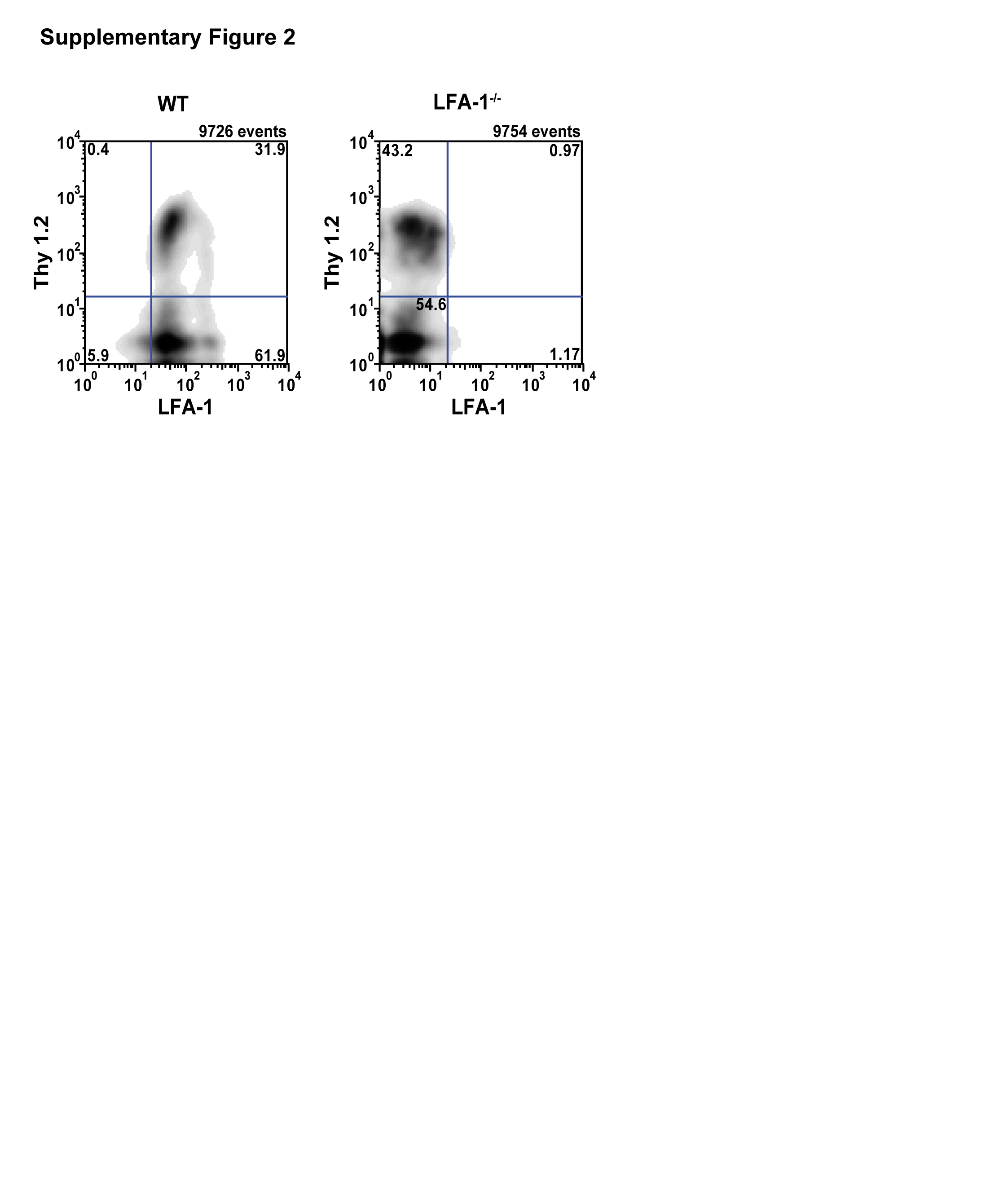

Supplement: S2 Fig — Quantification of Thy1.2+ T cells within the blood of untreated WT or LFA-1-/- mice using flow cytometry. A representative assay is shown (n = 3). (TIF) [file pone.0168001.s002.tif]

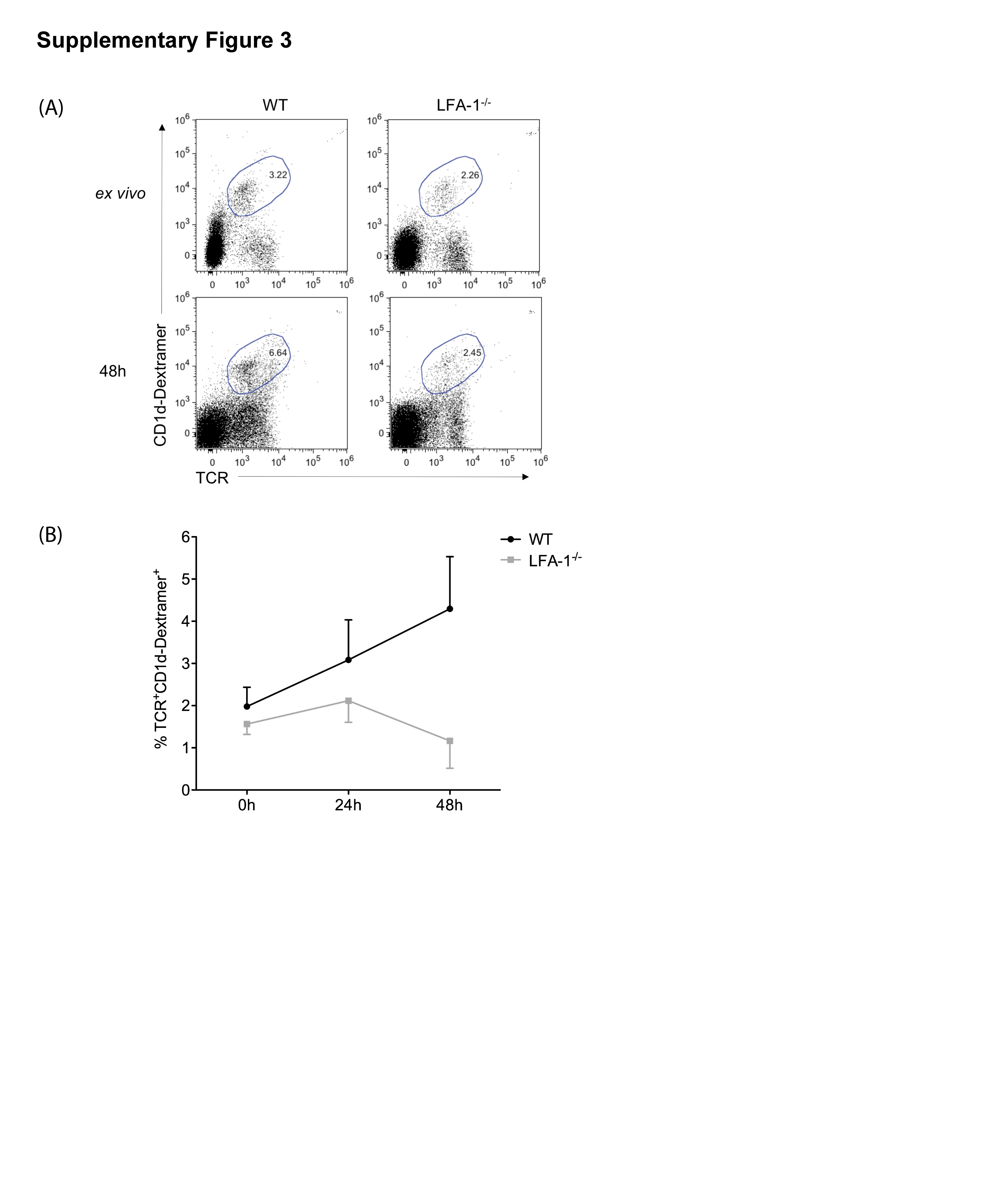

Supplement: S3 Fig — (A) Detection of TCRß+αGalCer/CD1d-dextramer+ cells within the liver of WT or LFA-1-/- mice before (ex vivo) or 48h post PH and LPS using flow cytometry. One representative assay is shown; 20.000 events per dot plot are shown. (B) Quantification of TCRß+αGalCer/CD1d-dextramer+ NKT cell staining depicted in (A); 0h: n = 4, 24h: n = 3, 48h: n = 3. (TIF) [file pone.0168001.s003.tif]
